# Response to ‘Performance of large language models at the MRCS Part A: a tool for medical education?’

**DOI:** 10.1308/rcsann.2025.0067

**Published:** 2025-10-01

**Authors:** G Kourounis, SJ Tingle, C Wilson

**Affiliations:** ^1^ Newcastle University, UK; ^2^ NIHR Blood and Transplant Research Unit at Cambridge and Newcastle Universities, UK; ^3^ The Freeman Hospital, UK

Dear Editor,

We read with interest the recent article by Yiu and Lam, ‘Performance of large language models at the MRCS Part A: a tool for medical education?’, published in the July 2025 issue of the *Annals.*^1^ This follows the May 2025 issue of the *Bulletin*, which was dedicated to artificial intelligence (AI) technologies in surgery. The topic is of clear ongoing interest for the college,^2,3^ and we would like to offer our reflections.

The authors have rightly identified a central question facing the surgical education community: can emerging AI technologies, particularly large language models (LLMs), be integrated reliably as educational tools? The results are of course impressive, with ChatGPT-4 achieving a score sufficient to pass the MRCS Part A examination. However, this achievement is tempered by the finding that these models produced inaccurate but authoritative answers for 15 to 36 percent of questions, depending on the platform assessed.

Although there are no data available on the performance of the latest LLM models on the MRCS Part A, it is reasonable to anticipate some degree of error. Even if the error rate were reduced to around 5 percent, this would still result in a significant number of incorrect responses given the large volume of questions typically attempted in preparation for this examination. In practical terms, a candidate using these tools as a learning resource would be exposed to incorrect information at a rate that cannot be overlooked. This is particularly concerning given the confident tone with which LLMs tend to deliver their responses. To echo the authors’ question: a tool for medical education? On balance, perhaps we are not quite there yet.

This article also highlights a subtle but important difference between AI-supported clinicians and clinicians who have been educated with the help of AI-enabled tools. These tools can help make the large and challenging volume of material an aspiring surgeon must learn more engaging and memorable. LLMs could help identify areas of weakness, put together bespoke curricula to address gaps, and individualise learning.^4^ Establishing the performance of LLMs on the MRCS Part A is an important and necessary first step, but further research is needed to establish their utility in surgical education. To use a chess analogy, it is one thing to build Deep Blue and beat Garry Kasparov; it is another to use Deep Blue to train the next generation of chess players so that they too beat Kasparov.

We commend the authors for their contribution to this area. Their work provides a valuable foundation for future research exploring the safe and effective incorporation of LLMs into surgical education. As this field progresses, careful evaluation and ongoing scrutiny will be essential to ensure that these technologies enhance, rather than undermine, the standards of surgical training. Ultimately, our commitment remains to improving patient care and any advancement in educational technology should be measured by its ability to bring us closer to this goal.

## Disclosure

No specific funding has been received for this work. GK has received funding via the National Institute for Health and Care Research (NIHR) Blood and Transplant Research Unit in Organ Donation and Transplantation (NIHR203332)—a partnership between NHS Blood and Transplant, University of Cambridge and Newcastle University. SJT has received funding via a Medical Research Council (MRC) Clinical Research Training Fellowship (MRC/Y000676/1), which was part funded by Kidney Research UK. The views expressed are those of the author(s) and not necessarily those of the NIHR, NHS Blood and Transplant, MRC or the Department of Health and Social Care.
